# Hybrid two-stage active contour method with region and edge information for intensity inhomogeneous image segmentation

**DOI:** 10.1371/journal.pone.0191827

**Published:** 2018-01-29

**Authors:** Shafiullah Soomro, Asad Munir, Kwang Nam Choi

**Affiliations:** Department of Computer Science and Engineering, Chung-Ang University, Seoul 156-756, Republic of Korea; Beijing University of Technology, CHINA

## Abstract

This paper presents a novel two-stage image segmentation method using an edge scaled energy functional based on local and global information for intensity inhomogeneous image segmentation. In the first stage, we integrate global intensity term with a geodesic edge term, which produces a preliminary rough segmentation result. Thereafter, by taking final contour of the first stage as initial contour, we begin second stage segmentation process by integrating local intensity term with geodesic edge term to get final segmentation result. Due to the suitable initialization from the first stage, the second stage precisely achieves desirable segmentation result for inhomogeneous image segmentation. Two stage segmentation technique not only increases the accuracy but also eliminates the problem of initial contour existed in traditional local segmentation methods. The energy function of the proposed method uses both global and local terms incorporated with compacted geodesic edge term in an additive fashion which uses image gradient information to delineate obscured boundaries of objects inside an image. A Gaussian kernel is adapted for the regularization of the level set function and to avoid an expensive re-initialization. The experiments were carried out on synthetic and real images. Quantitative validations were performed on Multimodal Brain Tumor Image Segmentation Benchmark (BRATS) 2015 and *PH*^2^ skin lesion database. The visual and quantitative comparisons will demonstrate the efficiency of the proposed method.

## Introduction

Image segmentation is the well-defined scheme of partitioning an image into several regions and it has a great significance in image processing and computer vision [1]. Intensity variations and inhomogeneities often occur in medical images mostly due to the imperfect image acquisition process and influence of the external surroundings. The existence of inhomogeneities and complicated boundaries create difficulties in image segmentation and eventually, it leads to uncertainty for doctors and radiologists during the decision-making process. Active contour models have been popular techniques for those type of images [2]. These methods provide smooth contours for image segmentation by using some energy minimization principle [3].

Existing active contour models are mainly categorized as edge-based [3–8] and region-based [9, 10, 12–15] methods. Edge-based methods exploit image gradient information to deploy a balloon force used to capture object boundaries in curve evolution [5]. Nevertheless, these methods are very sensitive to noise and unable to segment objects in the presence of fuzzy or blurred boundaries. Alternatively, region-based methods essentially intend to distinguish each region by using the statistical and curvature information to control the movement of contour, thus they have better execution over noisy and blurred images. These methods are further categorized into a global [9–12] and a local [13–16] region based active contour methods.

**Fig 1:**
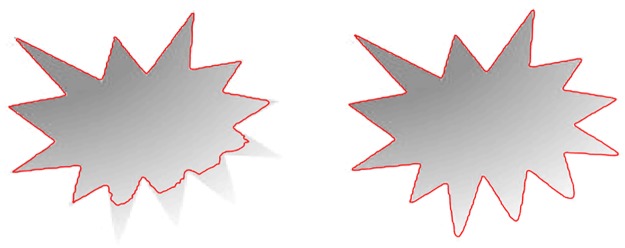
Intensity inhomogeneous image segmentation using. a: Chan-Vese method segmentation. b: LBF method segmentation.

One of the most popular global region method is Chan-Vese method [12], which is a special case of a piecewise constant Mumford and Shah method [10]. This method relies on constant intensity information across the regions therefore, it fails to segment images with intensity inhomogeneity. In order to overcome the limitation of the piecewise constant methods, Li et al. [14] proposed a local region-based method. This method has introduced a kernel function that considers the local image information known as LBF (local binary fitting) energy to segment images with intensity inhomogeneity. Segmentation of inhomogeneous image is shown in Fig 1, where Fig 1(a) shows segmentation of traditional Chan-Vese (global region method) and Fig 1(b) shows segmentation of LBF (local region method). From Fig 1, it can be deduced that LBF method is capable of handling intensity variations across the regions and give better result in terms of intensity inhomogeneity.

Recently, hybrid models [2, 17–22] have gained popularity among region based methods. These methods either combine region (local and/or global) and edge information or both local and global intensity information in their energy formulations. In [17], Zhang et al. proposed a method which combines the advantages of an edge-based and region-based active contours. It defines a SPF (signed pressure force) function using global means from Chan-Vese [12] method. A Gaussian kernel is also adapted to regularize level set and to avoid re-initialization. Oh et al. [20] has also formulated a hybrid method using scaled edge and region information. It combines global region information from Chan-Vese [12] method and coupled geodesic edge term from GAC [5] method to prevent boundary leakage problem.

Alternatively, A region-based active contour methods are proposed [22, 23] in the context of local and global intensity information. These methods integrate local and global intensity information in their energy formulations. Global force drives the movement of contour while local force captures the inhomogeneities across the regions.

A variational two-stage image segmentation approach for intensity inhomogeneity is proposed by Wang et al. [21]. Two stages of this method were based on global and local region active contours described by Gaussian distributions. The final segmentation of the first (global) stage is served as the initial contour of the second (local) stage. In this way, this method is able to segment images corrupted by intensity inhomogeneity.

This paper presents, a novel two-stage hybrid edge and region(local and global) active contour method for image segmentation with a level set formulation. Firstly, we obtain the coarse segmentation result of the first stage and the final segmentation result in the second stage. In the first stage, we incorporate global region force term and geodesic edge term for statistical and edge information. Global intensity used to segment homogeneous regions while edge term used to prevent boundary leakage problem. In the second stage, we develop a modified energy function which integrates local binary fitting term and geodesic edge term. Minimization of an energy functional in the second stage mutually segments the objects with intensity inhomogeneity and restrict the contour at object boundary. Since the local fitting methods [14, 15] are very sensitive to the initial position of contour. Proposed method takes final contour of the first stage as initialization of the second stage thus, it provides a better choice for initial contour position and give satisfactory performance for intensity inhomogeneous image segmentation.

In level set methods, it is often required to initialize the level set function to SDF (signed distance function) and avoid it to be too steep or too flat near the interface. In this regard, re-initialization is required to maintain the stability of level set function. There are numerous operational methods to implement re-initialization schemes. Traditional variants of the level set piecewise constant methods [11, 24, 25] eliminate the re-initialization by solving the constrained optimization problems. Liu et al. [26] proposed Lagrangian constrained optimization method to eliminate the re-initialization in level set function.

Proposed method adapts a Gaussian kernel from Zhang et al. [17] which not only regularizes the level set but also avoid an expensive re-initialization. Proposed method has been executed over several real and synthetic images including brain and skin lesion datasets and compared with previous methods. Experimental results will determine that proposed method offers an improved scheme and achieve better segmentation results than previous methods.

Rest of this paper is organized as follows. The background study is discussed in section 2. The proposed method is explained in section 3. Final results and comparisons are illustrated in section 4 using both real and synthetic images. The quantitative results are shown in section 5 using the public dataset from Multimodal brain tumor image segmentation benchmark (BRATS) 2015 [27] and *PH*^2^ skin lesion [28] database. In the end, conclusion and future work are described in section 6.

## Background and theoretical justifications

### Geodesic active contour model

GAC method [5] is regarded as a standard active contour method which uses image gradient information from the boundary of the object. Let *I*: Ω ⊂ *R*^2^ is an image domain, *I*: Ω → *R* is an input image and *C(q)* is a closed curve. They proposed the following energy functional:

$$\begin{array}{c} E_{GAC}\left(C\left(q\right)\right)=\int_{0}^{1}g\left(|\nabla I\left(C\left(q\right)\right)|\right)C^{\prime }\left(q\right)dq, \end{array}$$ (1)

where *g* is the edge stopping function defined as:

$$\begin{array}{c} g(\nabla I)=\frac{1}{1+|\nabla G_{\sigma }*I|} \end{array}$$ (2)

where ∇*G*_*σ*_ * *I* is the convolution of an image *I* with a Gaussian kernel whose standard deviation is *σ*. By minimizing the above energy functional we get following Euler–Lagrange equation:

$$\begin{array}{c} C_{t}=g\left(|\nabla I|\right)k\vec{N}-\left(\nabla g.\vec{N}\right)\vec{N} \end{array}$$ (3)

where *k* denotes the curvature of the contour and $\vec{N}$ is inward normal of the curve. The final level set equation is defined as follows:

$$\begin{array}{c} \frac{\partial \phi }{\partial t}=gdiv(\frac{\nabla \phi }{\left|\nabla \phi \right|})\left|\nabla \phi \right|+\nabla g.\nabla \phi \end{array}$$ (4)

This method relies on edge based contour evolution which can only capture objects with edges defined by its gradient. Therefore, this method doesn’t support regional information and fall into local minimum when the initial contour is not placed near object boundaries.

### Mumford and Shah model

Mumford and Shah [10] proposed a region-based image segmentation method. This method finds an optimal piecewise approximation function *μ* of the image *I*, which keep changing smoothly within a sub-region of image domain *I*: Ω ⊂ *R*^2^. The proposed energy formulation of this method is written as:

$$\begin{array}{c} E_{MS}=\lambda \underset{\Omega }{\int }{\left(I\left(x\right)-\mu \left(x\right)\right)}^{2}+v\underset{\Omega \backslash C}{\int }{\left(\nabla \mu \left(x\right)\right)}^{2}dx+\mu L\left(C\right) \end{array}$$ (5)

where *L*(*C*) is the length of the curve, *μ* and *v* are positive parameters. Practically, the energy functional of this model owns non-convex behavior and the non-regularity of the edge term causes difficulty during energy minimization.

### Chan-Vese model

In [12], Chan-Vese proposed a simplified energy functional based on Mumford and Shah model [10] by approximating the image intensities inside and outside of contour known as *c*_1_ and *c*_2_ respectively. Let *I*: Ω ⊂ *R*^2^ be an input image, *ϕ*: Ω ⊂ *R*^2^ a level set function and *C* a closed curve corresponding to the zero level set: *C* = {*x* ∈ Ω|*ϕ*(*x*) = 0}. The energy functional of Chan-Vese method is defined as follows:

$$\begin{array}{ll} E_{C}{}_{V}\left(C,c_{1},c_{2}\right) & =\lambda_{1}\underset{\Omega }{\int }{\left|I\left(x\right)-c_{1}\right|}^{2}H_{\varepsilon }\left(\phi \left(x\right)\right)dx+\lambda_{2}\underset{\Omega }{\int }{\left|I\left(x\right)-c_{2}\right|}^{2}\left(1-H_{\varepsilon }\left(\phi \left(x\right)\right)\right)dx\\ & +\mu \underset{\Omega }{\int }{\left|\nabla H_{\varepsilon }\left(\phi \right)\right|}^{2}dx+v\underset{\Omega }{\int }H_{\varepsilon }\left(\phi \right)dx \end{array}$$ (6)

where *μ* ≥ 0, *v* ≥ 0 and λ_1_, λ_2_ ≥ 0 are scaling constants, constant *μ* ≥ 0 scales the Euclidian length of the curve *C* and constant *v* scales the area term inside the contour *C*. *H*_*ε*_(*ϕ*) is the regularized Heaviside function defined as:

$$\begin{array}{c} H_{\varepsilon }\left(\phi \right)=\frac{1}{2}(1+\frac{2}{\pi }\arctan(\frac{\phi }{\varepsilon })) \end{array}$$ (7)

where *ϵ* handles the smoothness of Heaviside function. In Eq (6), two constants *c*_1_ and *c*_2_, represent global region intensities inside and outside of contour *C*, respectively. Minimizing Eq (6), with respect to *ϕ* using gradient descent method [29], the corresponding level set formulation is obtained as follows.

$$\begin{array}{c} \frac{\partial \phi }{\partial t}=\left(-\lambda_{1}{\left(I-c_{1}\right)}^{2}+\lambda_{2}{\left(I-c_{2}\right)}^{2}+\mu div\left(\frac{\nabla \phi }{\left|\nabla \phi \right|}\right)-v\right)\delta_{\varepsilon }\left(\phi \right) \end{array}$$ (8)

*δ*_*ϵ*_(*ϕ*) is a smooth version of a Dirac delta function, which is defined as:

$$\begin{array}{c} \delta_{\varepsilon }\left(\phi \right)=\frac{\varepsilon }{\pi (\phi^{2}+\varepsilon^{2})} \end{array}$$ (9)

beside handling the smoothness of a Heaviside function in Eq (7), *ϵ* also controls the width of the a Dirac delta function in Eq (9). Keeping *ϕ* fixed and minimizing energy function in Eq (6), with respect to *c*_1_ and *c*_2_, we have:

$$\begin{array}{c} c_{1}=\frac{\int_{\Omega }I(x)H_{\varepsilon }\left(\phi \left(x\right)\right)dx}{\int_{\Omega }H_{\varepsilon }\left(\phi \left(x\right)\right)dx} \end{array}$$ (10)

$$\begin{array}{c} c_{2}=\frac{\int_{\Omega }I(x)\left(1-H_{\varepsilon }\left(\phi \left(x\right)\right)\right)dx}{\int_{\Omega }(1-H_{\varepsilon }\left(\phi \left(x\right)\right))dx} \end{array}$$ (11)

The curve evolution process in Chan-Vese method is related to global characteristic of an image region inside and outside curve *C*, respectively. Therefore, this method produces unacceptable result if the image has local or inhomogeneous regions inside and outside of the curve *C*.

### Local binary fitted model

In, [14] Li et al. proposed local binary fitted (LBF) method by encapsulating local image information into their energy functional to deal with intensity inhomogeneity problem. Let, *I*: Ω ⊂ *R*^2^ be an input image, *ϕ*: Ω ⊂ *R*^2^ is a level set function, and *C* is a closed curve. They propose following energy formulation:

$$\begin{array}{c} \begin{array}{cc} E_{LBF}\left(C,f_{1},f_{2}\right)= & \lambda_{1}\int_{\Omega }K_{\sigma }\left(x-y\right){\left|I\left(y\right)-f_{1}\left(x\right)\right|}^{2}H_{\epsilon }\left(\phi \left(y\right)\right)dy\\ & +\lambda_{2}\int_{\Omega }K_{\sigma }\left(x-y\right){\left|I\left(y\right)-f_{2}\left(x\right)\right|}^{2}\left(1-H_{\epsilon }\left(\phi \left(y\right)\right)\right)dy \end{array} \end{array}$$ (12)

where λ_1_, λ_2_ ≥ 0 are positive parameters, *H*_*ε*_(*ϕ*) is the regularized Heaviside function defined in Eq (7). *f*_1_(*x*) and *f*_2_(*x*) are locally approximated intensities inside and outside of contour *C* defined as.

$$\begin{array}{c} f_{1}\left(x\right)=\frac{K_{\sigma }*\left[H_{\epsilon }\left(\phi \right)I\left(x\right)\right]}{K_{\sigma }*H_{\epsilon }\left(\phi \right)} \end{array}$$ (13)

$$\begin{array}{c} f_{2}\left(x\right)=\frac{K_{\sigma }*\left[\left(1-H_{\epsilon }\left(\phi \right)\right)I\left(x\right)\right]}{K_{\sigma }*\left(1-H_{\epsilon }\left(\phi \right)\right)} \end{array}$$ (14)

In order to ensure stable solution, the distance regularization term from [4] is fused in to Eq (12). Moreover, the Euclidean length term is also added to regularize the zero curve of *ϕ*. The total variational formulation of this method is defined as:

$$\begin{array}{ll} \frac{\partial \phi }{\partial t} & =-\delta_{\varepsilon }\left(\phi \right)\left(\lambda_{1}\underset{\Omega }{\int }K_{\sigma }\left(x-y\right)|I\left(x\right)-f_{1}\left(y\right){|}^{2}dy-\lambda_{2}\underset{\Omega }{\int }K_{\sigma }\left(x-y\right){\left|I\left(x\right)-f_{2}\left(y\right)\right|}^{2}dy\right)\\ & +v\delta_{\varepsilon }\left(\phi \right)div\left(\frac{\nabla \left(\phi \right)}{\left|\nabla \left(\phi \right)\right|}\right)+\mu \left(\Delta \phi -div\left(\frac{\nabla \phi }{\left|\nabla \phi \right|}\right)\right) \end{array}$$ (15)

where *μ* is a scaling parameter for distance regularized penalty term and *v* is scaling parameter for length term which drives the movement of contour towards object boundaries. *K*_*σ*_ is a Gaussian kernel with a standard deviation which is defined as:

$$\begin{array}{c} K_{\sigma }\left(x-y\right)=\frac{1}{{\left(2\pi \right)}^{n/2}\sigma^{n}}\exp(-\frac{{\left|x-y\right|}^{2}}{2\sigma^{2}}) \end{array}$$ (16)

where *σ* is a standard deviation of the Gaussian kernel function which controls the degree of localization from small neighborhood to whole image domain.

The introduction of Gaussian kernel function considers an image local information inside and outside of contour *C* and enable this method to segment images with intensity inhomogeneity. however, local image information is not always sufficient to carry out an accurate segmentation. Moreover, this method is extremely sensitive to the position of initial contour and stuck into local minima if we place initial contour far from boundaries.

### ESRAC model

Oh et al. [20] proposed a hybrid region and edge method known as edge scaled region-based active contour method. This method redefines Chan-Vese [12] region information weighted by edge stopping function and integrate geodesic edge term [5] into their formulation. They propose following energy formulation:

$$\begin{array}{ll} E_{ESRAC} & =\alpha \left(\underset{\Omega }{\int }g\left(I\left(x\right)\right)|I\left(x\right)-c_{1}{|}^{2}dx+\underset{\Omega }{\int }g\left(I\left(x\right)\right){\left|I\left(x\right)-c_{2}\right|}^{2}dx\right)\\ & +\left(1-\alpha \right)\overset{1}{\underset{0}{\int }}g\left(\left|\nabla I\left(C\left(q\right)\right)\right|\right)C^{\prime }\left(q\right)dq+v\underset{\Omega }{\int }dx \end{array}$$ (17)

where *g(x)* is an edge stopping function scaled with region force term. parameter *v* used to scale the area term inside and outside the contour *C* and *α* is scaling parameter whose values ranges between 0 ≤ *α* ≤ 1, it controls the balance between region and geodesic edge term. Minimizing Eq (17), using gradient descent method [29], the corresponding level set formulation is obtained as follows.

$$\begin{array}{c} \begin{array}{cc} \frac{\partial \phi }{\partial t}= & |\nabla \phi |[\alpha (-g\left(I\right){\left(I-c_{1}\right)}^{2}+g\left(I\right){\left(I-c_{2}\right)}^{2})\\ & +\left(1-\alpha \right)(g\left(I\right)div(\frac{\nabla \phi }{\left|\nabla \phi \right|})+\nabla g\left(I\right)\frac{\nabla \phi }{\left|\nabla \phi \right|})-v] \end{array} \end{array}$$ (18)

In above equation *g* is monotonically decreasing edge function defined as:

$$\begin{array}{c} g\left(I\left(x,y\right)\right)={\left(1-\lambda |\nabla G_{\sigma }*I\left(x,y\right){|}_{\infty }^{2}\right)}_{+}^{2} \end{array}$$ (19)

The regional term in above equation is weighted with edge indicator function therefore, this method can segment object boundaries by using gradient information. In the case of insufficient gradient information this method can also segment object by using statistical region information. However, this method is not available for inhomogeneous intensity regions.

## Proposed model

Inspired by the work of Oh et al. [20] and wang et al. [21] we propose a novel two-stage active contour segmentation method by integrating the region-based global and local methods with geodesic active contour method. In the first stage, we acquire segmentation result from the global region-edge active contour method (GREAC). This method rapidly captures all homogeneous regions and spurious boundaries in an image and achieves its coarse segmentation result. Later in the second stage, we use final contour of the last stage as initialization curve and continue segmentation process by minimizing the energy functional of the local region-edge active contour method (LREAC). The initial stage gives a partial segmentation result and provide a suitable initialization for second stage, in this way this method eliminates the common problem of initial contour existed in local region based methods [13, 14]. Finally, the second stage considers an image local information and gets the final segmentation result.

### First stage

Global region-based segmentation has been regarded as a fast and stable segmentation for homogeneous regions and it takes global image intensities inside and outside of contour. Furthermore, it is not sensitive to initialization of contour. In the first stage, we propose an energy functional of the global region-edge active contour method (GREAC) defined as:

$$\begin{array}{l} E_{GREAC}=\\ w\left(g\left(\left|\nabla I\right|\right)\underset{\Omega }{\int }{\left|I\left(x\right)-c_{1}\right|}^{2}H_{\varepsilon }\left(\phi \left(x\right)\right)dx+g\left(\left|\nabla I\right|\right)\underset{\Omega }{\int }{\left|I\left(x\right)-c_{2}\right|}^{2}\left(1-H_{\varepsilon }\left(\phi \left(x\right)\right)\right)dx\right)\\ \;+\left(1-w\right)\left(\overset{1}{\underset{0}{\int }}g\left(\left|\nabla I\left(C\left(q\right)\right)\right|\right)C^{\prime }\left(q\right)dq\right)+v\underset{\Omega }{\int }H_{\varepsilon }\left(\phi \right)dx \end{array}$$ (20)

In Eq (20), *c*_1_ and *c*_2_ are edge scaled intensity means defined as::

$$\begin{array}{c} c_{1}=\frac{\int_{\Omega }g(|\nabla I|)I(x)H_{\varepsilon }\left(\phi \left(x\right)\right)dx}{\int_{\Omega }g(|\nabla I|)H_{\varepsilon }\left(\phi \left(x\right)\right)dx} \end{array}$$ (21)

$$\begin{array}{c} c_{2}=\frac{\int_{\Omega }g(|\nabla I|)I(x)\left(1-H_{\varepsilon }\left(\phi \left(x\right)\right)\right)dx}{\int_{\Omega }g(|\nabla I|)(1-H_{\varepsilon }\left(\phi \left(x\right)\right))dx} \end{array}$$ (22)

where *g*(|∇*I*|) is a monotonically decreasing edge stopping function defined in Eq (2). By using steepest gradient descent method [29], minimization of Eq (20), with respect to *ϕ* leads to the following gradient decent flow:

$$\begin{array}{c} \begin{array}{cc} \frac{\partial \phi }{\partial t}= & |\nabla \phi |[w(-g\left(|\nabla I|\right){\left(I-c_{1}\right)}^{2}+g\left(|\nabla I|\right){\left(I-c_{2}\right)}^{2})\delta_{\varepsilon }\left(\phi \right)\\ & +\left(1-w\right)(g\left(|\nabla I|\right)div(\frac{\nabla \phi }{\left|\nabla \phi \right|})+\nabla g\left(I\right)\frac{\nabla \phi }{\left|\nabla \phi \right|})-v\delta_{\varepsilon }\left(\phi \right)] \end{array} \end{array}$$ (23)

**Fig 2:**
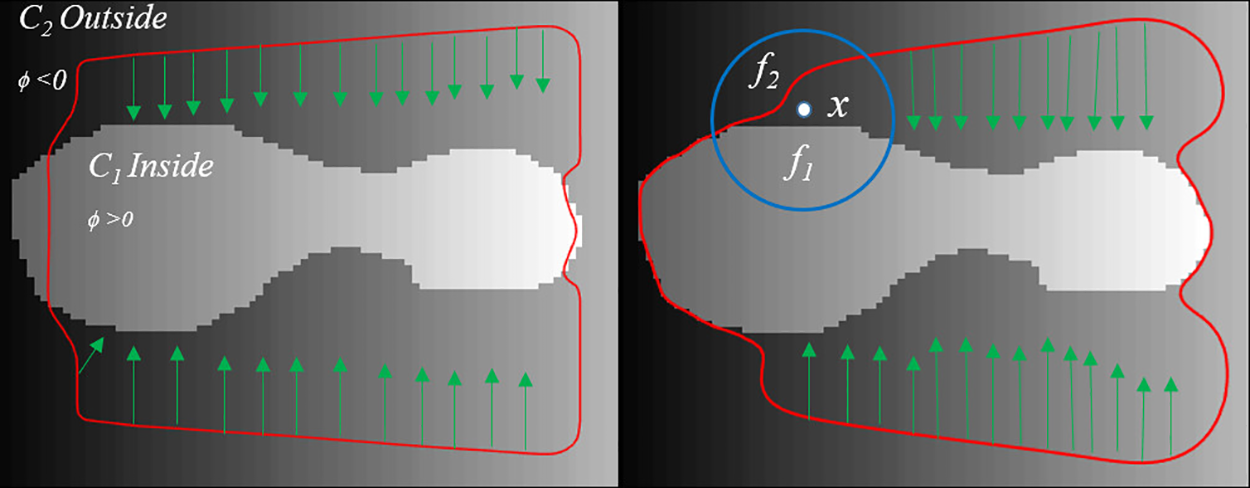
Graphical representation of taking intensity information, where *c*_1_, *c*_2_ are global and *f*_1_, *f*_2_ are local intensity means, green arrows represent the effect of geodesic term. a: First stage segmentation. b: second stage segmentation.

The energy functional in above equation is scaled with a parameter *w* whose value ranges between 0 ≤ *w* ≤ 1. This parameter manages the weight of the global region and geodesic edge term depending on the type of image we are dealing with. When *w* is large the global region term act as the main force with small influence of edge term to detect global regions and boundaries inside an object. Similarly, when *w* is small geodesic force term act as the main force with small influence of global region information to attract the curve towards the desired boundary of the object. This process is shown in Fig 2(a) which describes the role of global and geodesic energy terms during the first stage.

### Second stage

The first stage of the proposed method uses global image information across the regions. Therefore, it is unable to capture objects with intensity inhomogeneity. In this stage we extend global region-edge active contour method (GREAC) to local region-edge active contour method (LREAC) by using local binary fitting energy from LBF [14] method. The energy functional of the local region-edge active contour method (LREAC) is defined as:

$$\begin{array}{c} \begin{array}{cc} E_{LREAC}= & w(g\left(|\nabla I|\right)\int_{\Omega }K_{\sigma }\left(x-y\right){\left|I\left(y\right)-f_{1}\left(x\right)\right|}^{2}H_{\epsilon }\left(\phi \left(y\right)\right)dy\\ & \;+g\left(|\nabla I|\right)\int_{\Omega }K_{\sigma }\left(x-y\right){\left|I\left(y\right)-f_{2}\left(x\right)\right|}^{2}\left(1-H_{\epsilon }\left(\phi \left(y\right)\right)\right)dy)\\ & \;+\left(1-w\right)(\int_{0}^{1}g\left(|\nabla I\left(C\left(q\right)\right)|\right)C^{\prime }\left(q\right)dq)+v\int_{\Omega }H_{\varepsilon }\left(\phi \right)dx \end{array} \end{array}$$ (24)

*f*_1_(*x*) and *f*_2_(*x*) are locally approximated intensities inside and outside of contour *C* defined as.

$$\begin{array}{c} f_{1}\left(x\right)=\frac{K_{\sigma }*[H_{\epsilon }\left(\phi \right)g(|\nabla I|)I\left(x\right)]}{K_{\sigma }*H_{\epsilon }\left(\phi \right)g\left(|\nabla I|\right)} \end{array}$$ (25)

$$\begin{array}{c} f_{2}\left(x\right)=\frac{K_{\sigma }*[\left(1-H_{\epsilon }\left(\phi \right)\right)g(|\nabla I|)I\left(x\right)]}{K_{\sigma }*\left(1-H_{\epsilon }\left(\phi \right)\right)g\left(|\nabla I|\right)} \end{array}$$ (26)

where *g*(|∇*I*|) is a monotonically decreasing edge stopping function defined in Eq (2). By using steepest gradient descent method [29], minimization of Eq (24), with respect to *ϕ* leads to the following gradient decent flow:

$$\begin{array}{c} \begin{array}{cc} \frac{\partial \phi }{\partial t}= & |\nabla \phi |[-\delta_{\varepsilon }\left(\phi \right)w(g\left(|\nabla I|\right)\int_{\Omega }K_{\sigma }\left(x-y\right){\left|I\left(x\right)-f_{1}\left(y\right)\right|}^{2}dy\\ & -g\left(|\nabla I|\right)\int_{\Omega }K_{\sigma }\left(x-y\right){\left|I\left(x\right)-f_{2}\left(y\right)\right|}^{2}dy)\\ & +\left(1-w\right)(g\left(|\nabla I|\right)div(\frac{\nabla \phi }{\left|\nabla \phi \right|})+\nabla g\left(I\right)\frac{\nabla \phi }{\left|\nabla \phi \right|})+v\delta_{\varepsilon }\left(\phi \right)div(\frac{\nabla (\phi )}{\left|\nabla (\phi )\right|})] \end{array} \end{array}$$ (27)

The energy functional in above equation is also scaled with a parameter *w* whose value ranges between 0 ≤ *w* ≤ 1. This parameter manages the weight of the local region and geodesic edge term depending on the type of image we are dealing with. When the *w* is large the local-region term act as the main force with small influence of edge term to detect inhomogeneous regions and boundaries inside an object. Similarly, when *w* is small geodesic force term act as the main force with small influence of local-region information to attract the curve towards the desired boundary of the object. This process is shown in Fig 2(b) which describes the role of local and geodesic energy terms during the second stage.

Traditional let set methods need to initialize their level set function (*ϕ*) periodically to SDF (signed distance function) during the contour evolution. Li et al. [4] proposed a penalization term which is also computationally expensive. The proposed method uses a Gaussian filter which regularizes a level set function and also avoids an expensive re-initialization. The initial level set function *ϕ*_0_ for the proposed method is defined as:

$$\begin{array}{c} \phi \left(x,t=0\right)=\{\begin{array}{c} -\rho \;x\in \Omega \setminus \partial \Omega \\ 0\;x\in \partial \Omega \\ \rho \;x\in \Omega \setminus \Omega \end{array} \end{array}$$ (28)

where *ρ* ≥ 0 is a constant, Ω_0_ is region inside an initial contour, Ω is the image domain and ∂Ω is an initial contour. Finally, the iterative steps of the proposed method are summarized in following algorithm:

**Algorithm**

1. **Stage 1:** First stage segmentation via global region-edge active contour method.

2. **Initialize** *ϕ*, *ϕ*(*x*, *t* = 0) from Eq (28).

3. *n* = 1.

4. **while** *n* <*N*_*max*_ ***do***

5. Compute (2), (21) and (22), respectively.

6. Solve *ϕ* using (23).

7. *n* = *n* + 1.

8. **end while**

9. **Output:** Rough segmentation result, final $\phi^{N_{max}}$.

10. **Stage 2:** Final stage segmentation via Local region-edge active contour method.

11. **Initialize** *ϕ*, *ϕ*(*x*, *t* = 0) using last stationary level set *ϕ*, *ϕ* = *ϕ*_*N*_*max*__.

12. *n* = 1.

13. **while** the solution is not converged do

14. Compute (2), (25) and (26), respectively.

15. Solve *ϕ* using (27).

16. *n* = *n* + 1.

17. **end while**

18. **Output:** Final and accurate segmentation result, final *ϕ*.

## Results and quantitative comparisons

All the experiments were implemented in MATLAB on a personal computer with Intel core i5, 3.2 GHz, and 8 GB RAM. We have used parameters for all experiments which are listed in Table 1.

**Table 1:** Parameters used in the experiment and validation section.

Method	Force constant		Length term constant	Gaussian kernel constant	Initial level set constant	Dirac constant	Time-step	w constant
	λ_1_	λ_2_	*v*	*σ*	*ρ*	*ϵ*	Δ	*w*
**Chan-Vese** [12]	1	1	0	-	2	1	0.1	-
**LBF** [14]	1	1	0.001 * 255 * 255	3.0	2	1	0.1	-
**LCV** [16]	-	-	0.001	-	1	1	0.1	-
**Wang et al.** [21] **(1st stage)**	-	-	0.1 * 10^−6^ * 255^2^	3.0	2	1	0.2	-
**Wang et al.** [21] **(2nd stage)**	-	-	0.05 * 10^−7^ * 255^2^	3.0	2	1	0.05	-
**Proposed(1st stage)**	1	1	1	-	2	1	0.1	0.08
**Proposed(2nd stage)**	1	1	0.1 * 10^−4^	3	2	1	0.1	0.01

**Fig 3:**
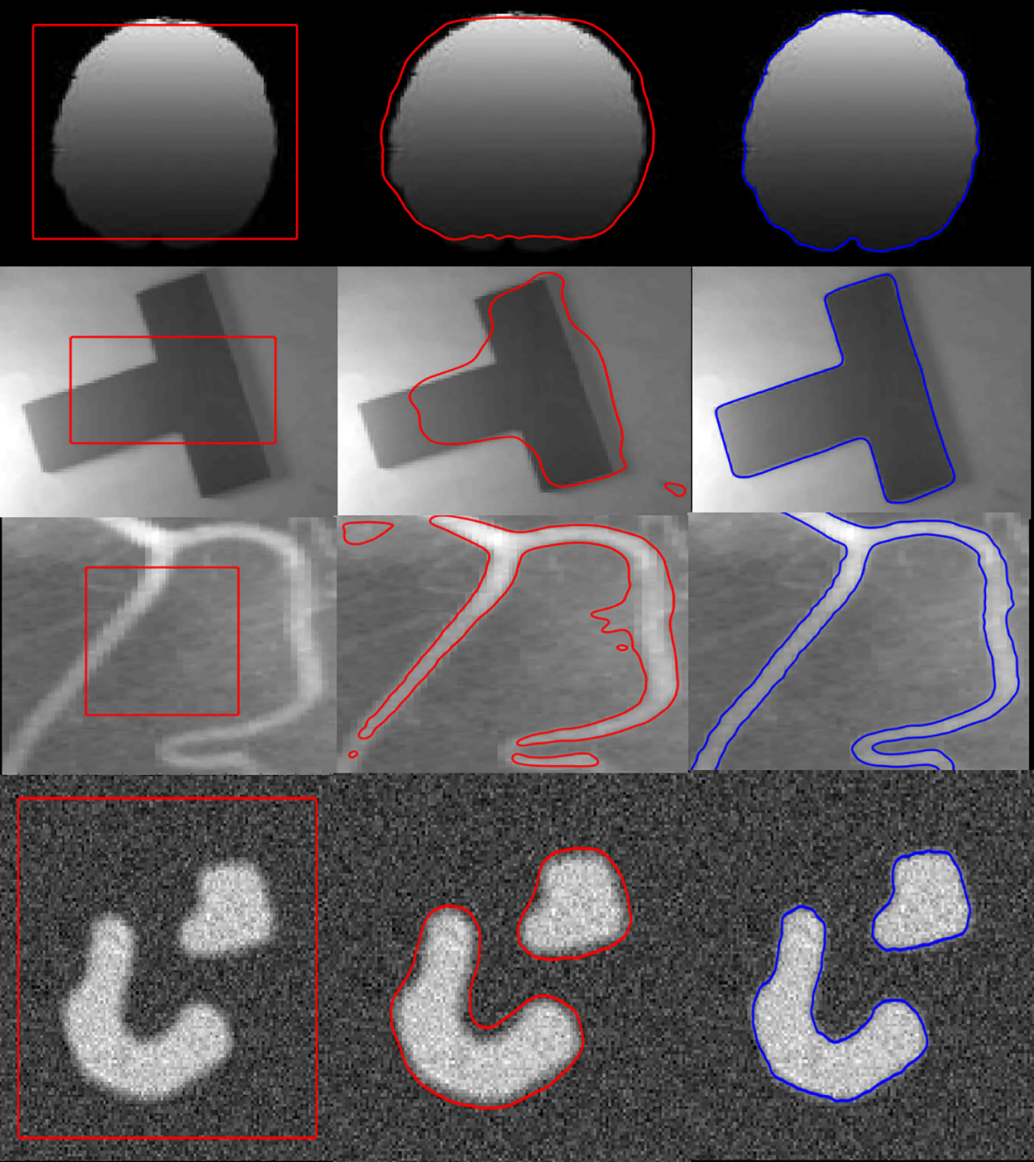
Segmentation using proposed method. (a): original images with initial contours. (b): first stage segmentation result after 10 iterations. (c): second stage segmentation result.

Fig 3 shows the first experiment of the proposed method on four synthetic images with intensity inhomogeneity. Column (a) show original images with initial contours, column (b) shows the result of the first stage and column (c) shows the final (second stage) segmentation result of the proposed method. Proposed method first produces rough segmentation result in column (b) which provides a suitable initialization for the second stage and thus it helps to get desired segmentation smoothly.

**Fig 4:**
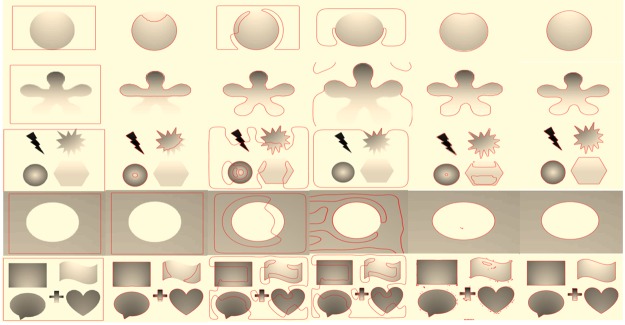
Segmentation results using images from different intensity variations. (a): original images with initial contours. (b): Chan-Vese result. (c): LBF result. (d): LCV result. (e): Wang et al. result. (f): proposed method result.

Fig 4 shows the result of the proposed method and its comparison with previous methods on five different synthetic images involving intensity inhomogeneity in foreground and background of the objects. The column (a) showing original images with initial contours, the column (b) shows the result of Chan-Vese [12] method and produce an unacceptable result because this method can only capture homogeneous intensity regions. Column (c) and (d) show the result of LBF [14] method and LCV [16] method since these methods are very sensitive to initial contour therefore, these methods have not properly delineated the required object boundary. Column (e) shows the result of Wang et al. [21] method. This method is able to properly segment the second image, however, some unwanted contour has been produced in a fourth and fifth image. Moreover, this method is unable to detect obscured edge in the first image. Column (f) shows the result of the proposed method which is able to capture and segment all the objects properly.

Table 2 illustrates the time complexity for Fig 4 in terms of CPU time and iteration. It shows that Chan-Vese [12] method has conceded least time and iterations but it has not achieved desired segmentation results. On the other proposed method has achieved an accurate segmentation results with less number of iterations and CPU time compared to LBF [14], LCV [16] and Wang et al. [21] methods respectively.

**Table 2:** CPU time and iterations consumed by each method in Fig 4.

Methods		Image 1	Image 2	Image 3	Image 4	Image 5
Chan-Vese [12]	Iterations	**20**	**20**	**20**	**20**	**20**
	CPU time(sec)	**3.27**	**2.96**	**4.27**	**3.48**	**4.65**
LBF [14]	Iterations	50	145	100	50	96
	CPU time(sec)	12.39	19.95	26.13	13.32	25.64
LCV [16]	Iterations	1000	1500	1500	1500	1500
	CPU time(sec)	80.36	80.90	83.89	88.13	84.36
Wang et al. [21]	Iterations	58	210	200	100	220
	CPU time(sec)	6.80	5.73	26.19	15.91	23.89
Proposed method	Iterations	40	60	35	30	26
	CPU time (sec)	5.68	5.30	5.84	6.65	5.91

The Local region based active contour methods [14, 16] are able to distinguish small changes between the background and the foreground. Therefore, they are suitable for intensity inhomogeneous images. However, there exists an intrinsic drawback of initial contour in local region based active contour methods. Therefore such methods are not sufficient for segmenting inhomogeneous information alone. Proposed method eliminates the initial contour problem by providing an ideal contour position in the first stage from global region based segmentation. In second stage local method gets the advantage of an ideal contour position and capture all inhomogeneous regions efficiently.

**Fig 5:**
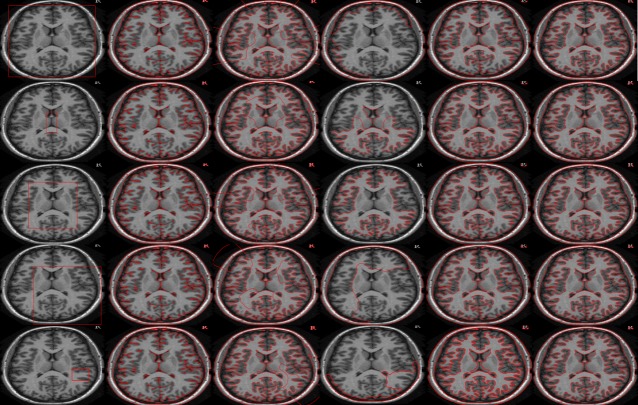
Segmentation results with different level set initialization. (a): original images with initial contours. (b): Chan-Vese result. (c): LBF result. (d): LCV result. (e): Wang et al. result. (f): Proposed method result.

In Fig 5, we have shown the influence of initial contour position on the segmentation result. Column (a) shows five different level set initializations on the same image, Chan-Vese [12] result is shown in column (a), LBF method [14] results are shown in column (c), LCV method [16] results are shown in column(d), Wang et al. [21] method results are shown in column (e) and proposed method results are shown in column (f) respectively. Results demonstrate that proposed method has not intruded by the position of initial contour, in contrast previous methods have shown their sensitivity to the initial contour and produced undesired results.

In Table 3, we have recorded the number of iterations and CPU time of each method based on Fig 5. The proposed method have conceded the second least number of iterations and CPU time after Chan-Vese [12] method and achieved desired segmentation results, whereas the Chan-Vese method was unable to get required results. Though, Wang et al. [21] method has also produced good results in the first and second row however it consumed a large number of iterations and CPU time for obtaining segmentation result, while proposed method has obtained the same results in all four initialization examples within less number of iterations and CPU time.

**Table 3:** CPU time(in seconds) and iterations consumed by each method in Fig 5.

Methods		row(1)	row(2)	row(3)	row(4)	row(5)
Chan-Vese [12]	Iterations	**20**	**20**	**20**	**20**	**20**
	CPU time(sec)	**4.31**	**2.75**	**2.78**	**3.41**	**3.20**
LBF [14]	Iterations	50	20	50	50	50
	CPU time(sec)	10.48	10.69	10.22	10.29	10.21
LCV [16]	Iterations	1500	1500	1500	1500	1500
	CPU time(sec)	30.21	38.450	38.90	39.46	38.04
Wang et al. [21]	Iterations	1000	200	1000	1000	800
	CPU time(sec)	22.88	6.058	24.10	23.24	18.63
Proposed method	Iterations	45	35	32	30	28
	CPU time (sec)	7.24	4.52	4.41	4.32	4.01

**Fig 6:**
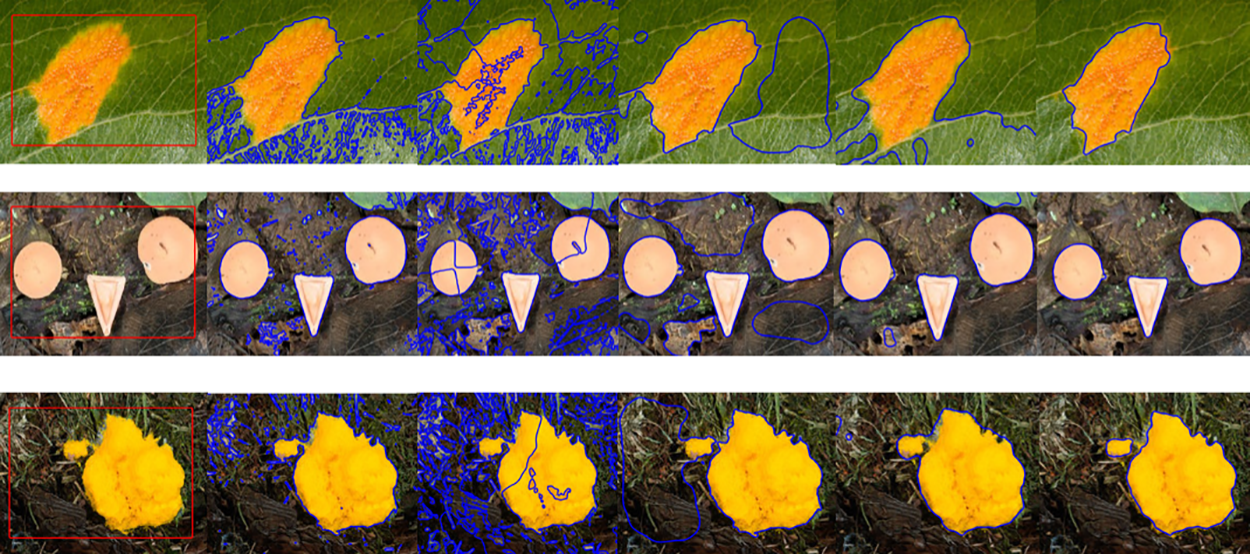
Segmentation results using real images. (a): Original images with initial contours. (b): Chan-Vese result. (c): LBF result. (d): LCV result. (e): Wang et al. result. (f): Proposed method result.

The experiment has also been performed on a real set of images with inhomogeneous and complex region properties. Fig 6 shows segmentation of three different real images, where column (a) shows the initial contour with original images. Column (b), (c), (d), (e) and (f) shows the result of Chan-Vese [12], LBF [14], LCV [16], Wang et al. [21] and proposed method respectively. All previous methods either capture uninterested regions or stuck in the background and thus have failed to get the desired segmentation results. On the contrary, proposed method gets the required results smoothly. Table 4 also shows the analysis of Fig 6 in terms of CPU time and iterations, where Chan-Vese method and Wang et al method yields the lowest time complexity analysis than proposed method but these methods have produced inappropriate segmentation results.

**Table 4:** CPU time(in seconds) and iterations consumed by each method in Fig 6.

Methods		row(1)	row(2)	row(3)
Chan-Vese [12]	Iterations	**20**	**20**	**20**
	CPU time(sec)	**2.72**	**3.21**	**2.89**
LBF [14]	Iterations	50	50	50
	CPU time(sec)	13.40	12.66	11.96
LCV [16]	Iterations	1500	1500	1500
	CPU time(sec)	31.17	31.13	33.12
Wang et al. [21]	Iterations	120	120	150
	CPU time(sec)	4.32	3.22	3.93
Proposed method	Iterations	60	50	55
	CPU time (sec)	7.02	6.52	6.74

**Fig 7:**
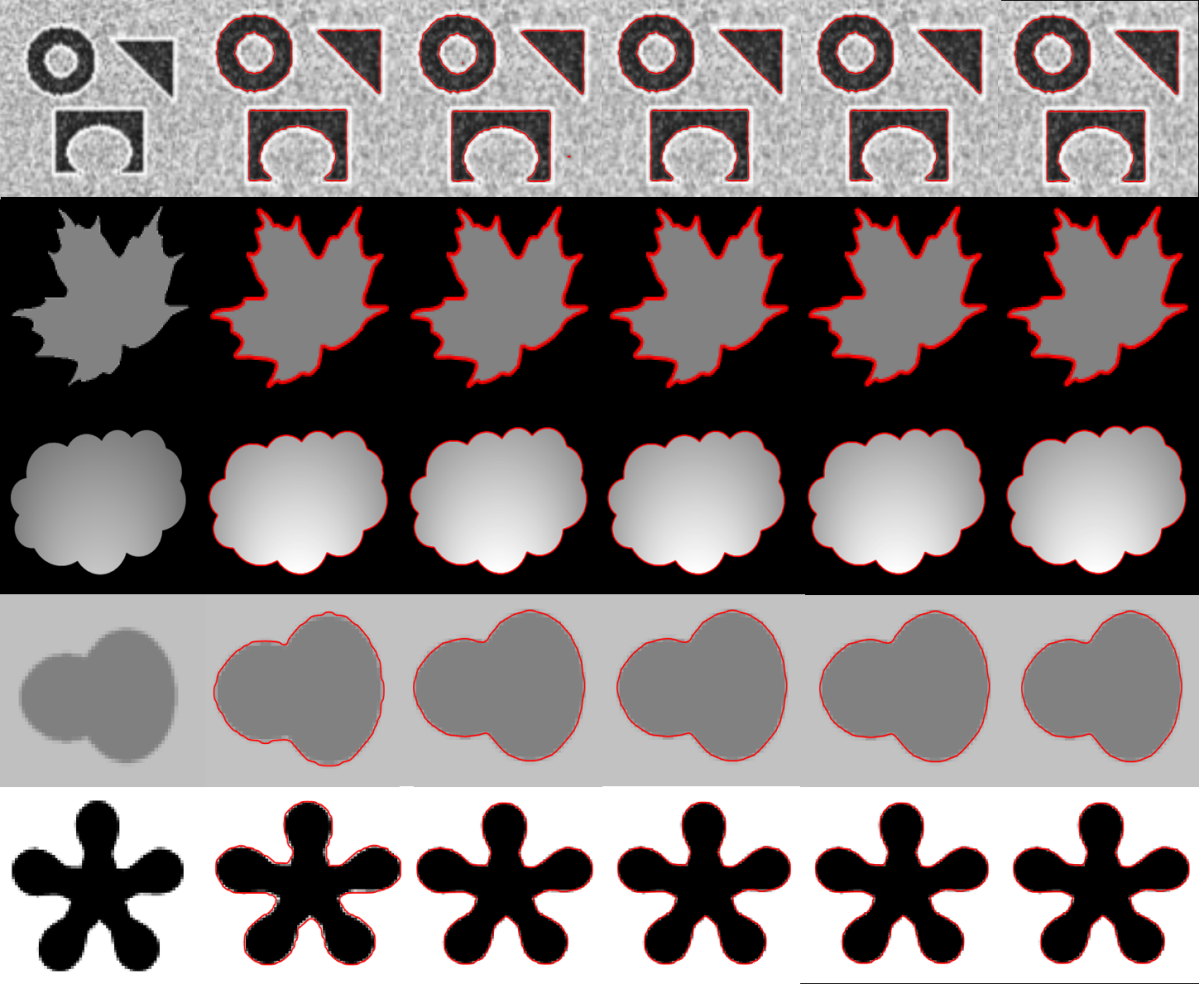
Segmentation results using the same accuracy level. Column(1): Original images. Column(2): Chan-Vese result. Column(3): LBF result. Column(4): LCV result. Column(5): Wang et al. result. Column(6): Proposed method result.

In order to compare the computational cost of the proposed method at same accuracy level with previous state-of-the-art methods, we have performed an experiment with five different images. Fig 7 shows approximately similar results obtained by all previous methods including proposed method. The first column in Fig 7 show original images followed by the result of the Chan-Vese [12] method, LBF [14] method, LCV [16] method, Wang et al. [21] method and proposed method in second, third, fourth, fifth and sixth column respectively. The CPU times of these images are listed in Table 5. By comparing the computational time of the proposed method and previous methods, it is clear that proposed method is also modest and proficient when it comes to same accuracy level with previous state-of-the-art methods.

**Table 5:** CPU time(in seconds) and iterations consumed by each method in Fig 7.

Methods		row(1)	row(2)	row(3)	row(4)	row(5)
Chan-Vese [12]	Iterations	20	15	30	50	20
	CPU time(sec)	1.82	**0.557**	**0.991**	4.06	1.356
LBF [14]	Iterations	20	10	15	25	10
	CPU time(sec)	6.859	2.794	3.972	17.557	3.704
LCV [16]	Iterations	200	100	130	300	100
	CPU time(sec)	14.731	2.664	2.881	28.956	3.221
Wang et al. [21]	Iterations	20	15	20	20	10
	CPU time(sec)	4.125	3.783	3.709	3.350	**1.305**
Proposed method	Iterations	10	10	15	10	10
	CPU time (sec)	**1.678**	1.508	1.009	**1.387**	1.467

## Quantitative analysis

The frequency of skin cancer has been consistently expanding over the past decade. Melanoma has been considered as one of the dangerous forms of skin cancer, reports show that survival rate of this cancer is very less during its last stage [30]. Therefore, detection of melanoma is very important and it helps dermatologists for initial treatment and diagnosis. Active contours have become a hot topic for melanoma image segmentation therefore, proposed method has been also validated over skin lesion dataset and compared with the previous state of art methods. In this regard, we use publically available skin lesion dataset *PH*^2^ [28] which comprises of 200 lesion images with manually annotated ground truths. The parameters used for skin lesion images are *v*1 = 0.0001 * 255 * 255, *v*2 = 0.00001 * 255 * 255, *σ* = 0.3, *w*1 = 0.9 and *w*2 = 0.001 respectively.

**Fig 8:**
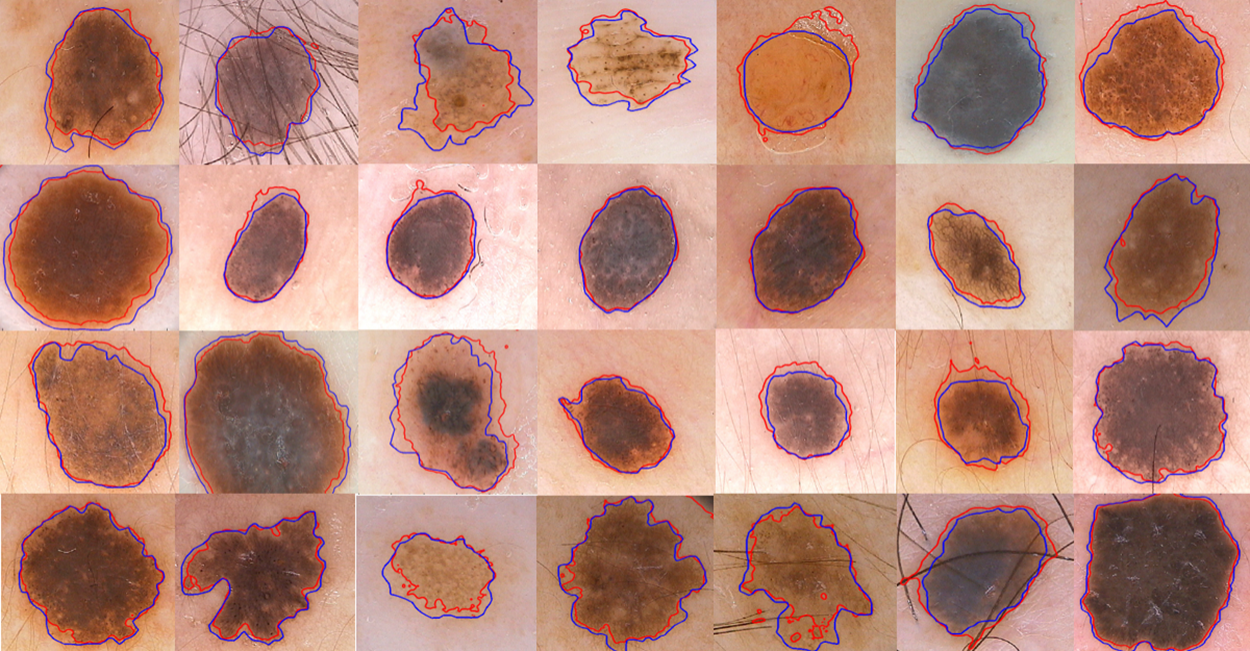
Segmentation results of proposed method (in red contour) with their respective ground truths (in blue contour).

**Fig 9:**
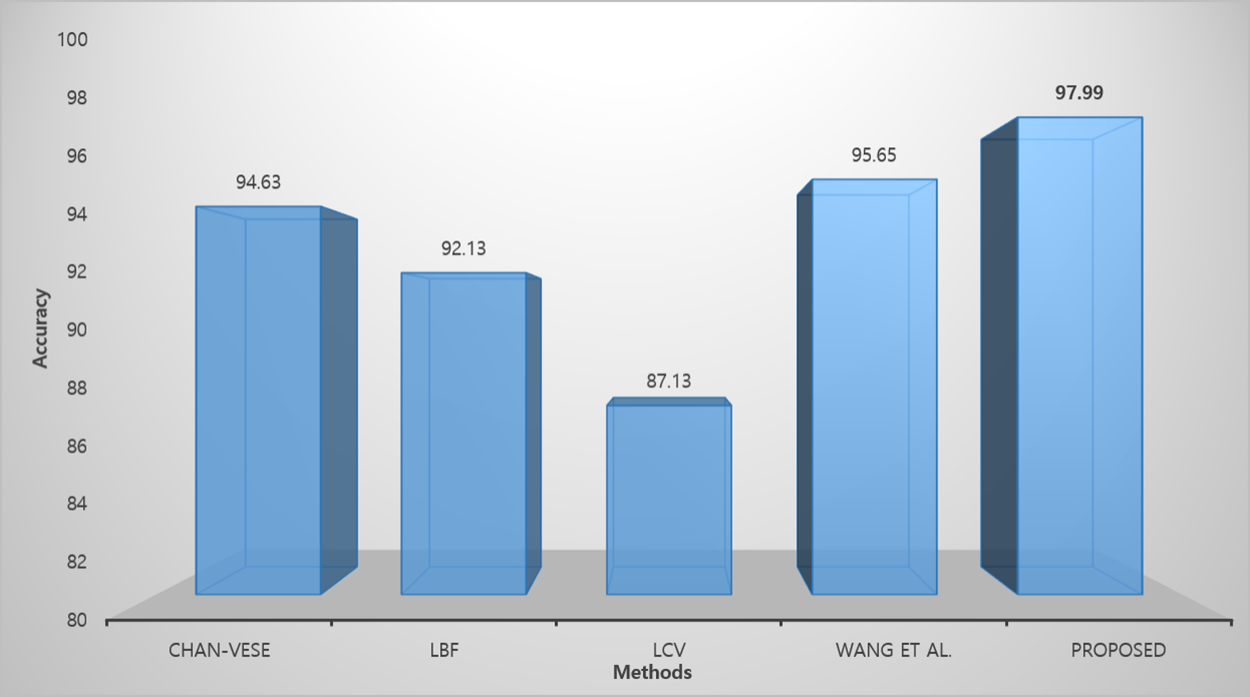
Segmentation accuracy of each method.

Fig 8 shows the result of proposed method on skin lesion images, where red contour shows the result of the proposed method and blue contour shows the ground truth respectively. Fig 9 shows the quantitative analysis of skin lesion segmentation in terms of accuracy analysis. We applied the proposed method and previous state of art methods on 200 skin images and calculated the average accuracy of each method. The formula for accuracy analysis is defined as:

$$\begin{array}{c} Accuracy=\frac{\left|A\cap B\right|}{\left|A\cup B\right|}\times 100, \end{array}$$ (29)

where A is the segmented region and B is the ground truth. Fig 9 shows that proposed method gets the high accuracy compared to the previous state of the art methods.

MRI (Magnetic Resonance Imaging) is regarded as most commonly used noninvasive modality for brain tumor detection [31]. Manual segmentation of brain tumor by radiologists and experts suffer from errors mostly due to tiredness and fatigue. Segmentation of abnormal regions in MRI is essential because it assists doctors and specialists to study the growth of a tumor and quantitatively analyze it over a period of time. Many segmentation approaches have been proposed for brain tumor segmentation. Recently active contours have gained much popularity among brain tumor segmentation [32]. Therefore, we have also validated our method on the BRATS 2015 challenge dataset [27]. Four MRI sequences are available for each patient known as: T1-weighted(T1), T1 with gadolinium-enhancing contrast (T1c), T2- weighted (T2) and FLAIR. We have tested our method on BRATS 2015 [27] training dataset which comprises of 200 HGG(high-grade glioma) and 44 LGG(low-grade) patient volumes. We use three validation metrics for quantitative evaluations which are Dice coefficient (DSC), sensitivity(Sen) and specificity (Spe). The obtained result will be considered as good when the measured values of these metrics are close to 1. Sensitivity metric value defines that all detected regions(tumors) are correct and belongs to the ground truth. similarly, specificity specifies that none of the healthy tissue is considered as tumor tissue. Moreover, Dice coefficient measure how much detected tumor region overlaps the ground truth. These metrics are defined as:

$$\begin{array}{c} DSC=\frac{2\times TP}{2\times TP+FP+FN} \end{array}$$ (30)

$$\begin{array}{c} Sen=\frac{TP}{TP+FN} \end{array}$$ (31)

$$\begin{array}{c} Spe=\frac{TN}{TN+FP} \end{array}$$ (32)

where TP (true positive)corresponds to segmented tumor tissues, TN (true negative) corresponds to correctly unsegmented regions, FP (false positive) corresponds to the normal regions considered as tumor regions and FN (false negative) corresponds to the undetected tumor regions, respectively. The proposed method parameters used for skin lesion images are *v*1 = 1, *v*2 = 0.0001 * 255 * 255, *σ* = 3, *w*1 = 0.6 and *w*2 = 0.751 respectively.

**Fig 10:**
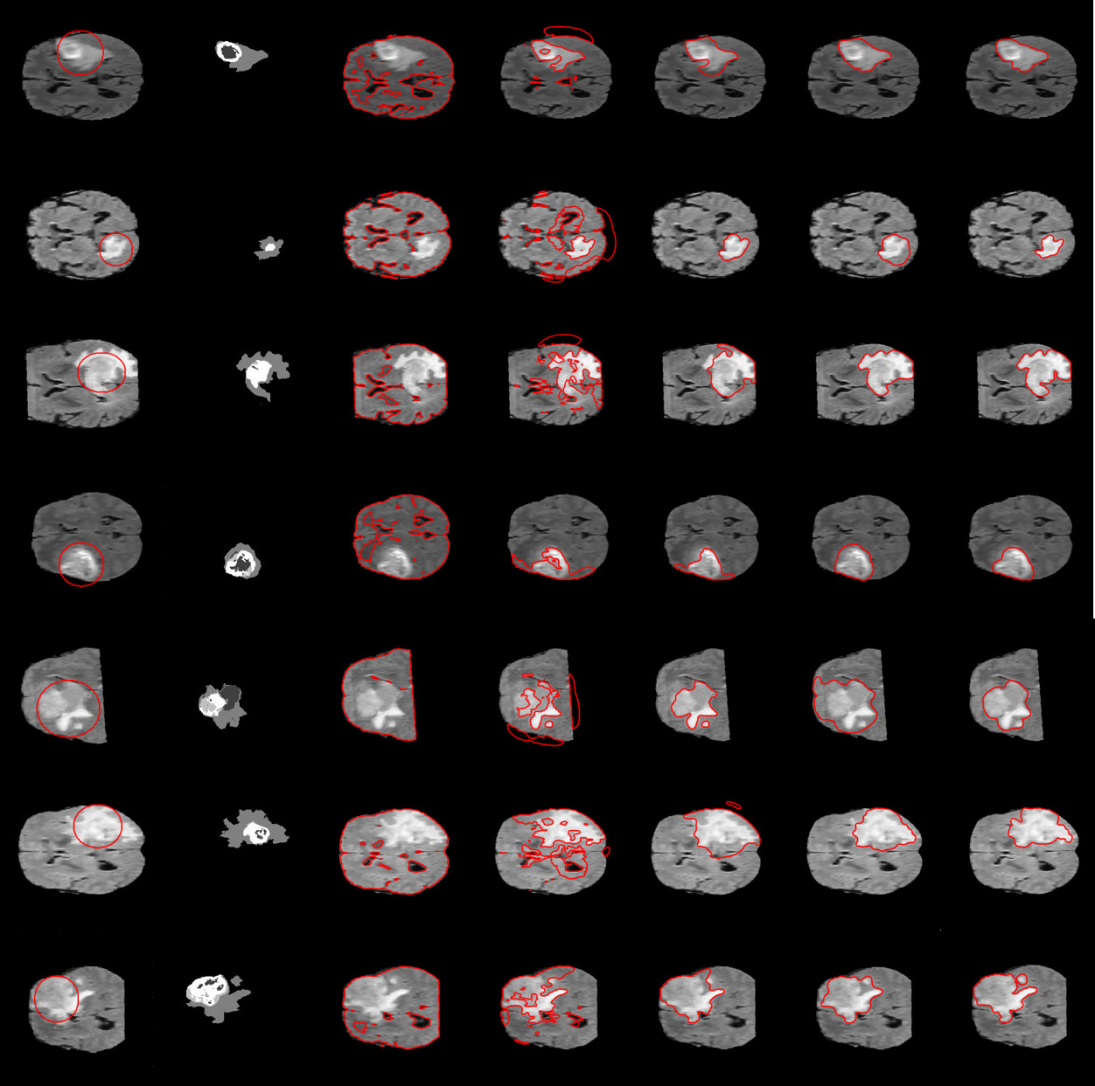
Segmentation results of the brain tumor: (From left to right) the Chan-Vese, LBF, LCV, wagn et al. and the proposed method.

Fig 10 presents obtained results on some images taken from BRATS 2015 [27]. The first column show the original images with initial contour and the second column shows the ground truth followed by the result of (from left to right) Chan-Vese [12], LBF [14], LCV [16], Wang et al. [21] and proposed method. It has be observed that final segmentation of the proposed method is robust and accurate against the complex background, where almost all previous methods fail to delineate the right boundaries. Moreover, Fig 10 show the segmentation results for three planes: axial, coronal and sagittal as shown in ground truth(second column). Results show that proposed method has accurately segmented brain tumor, Necrosis, and surrounding edema tissues successfully.

**Fig 11:**
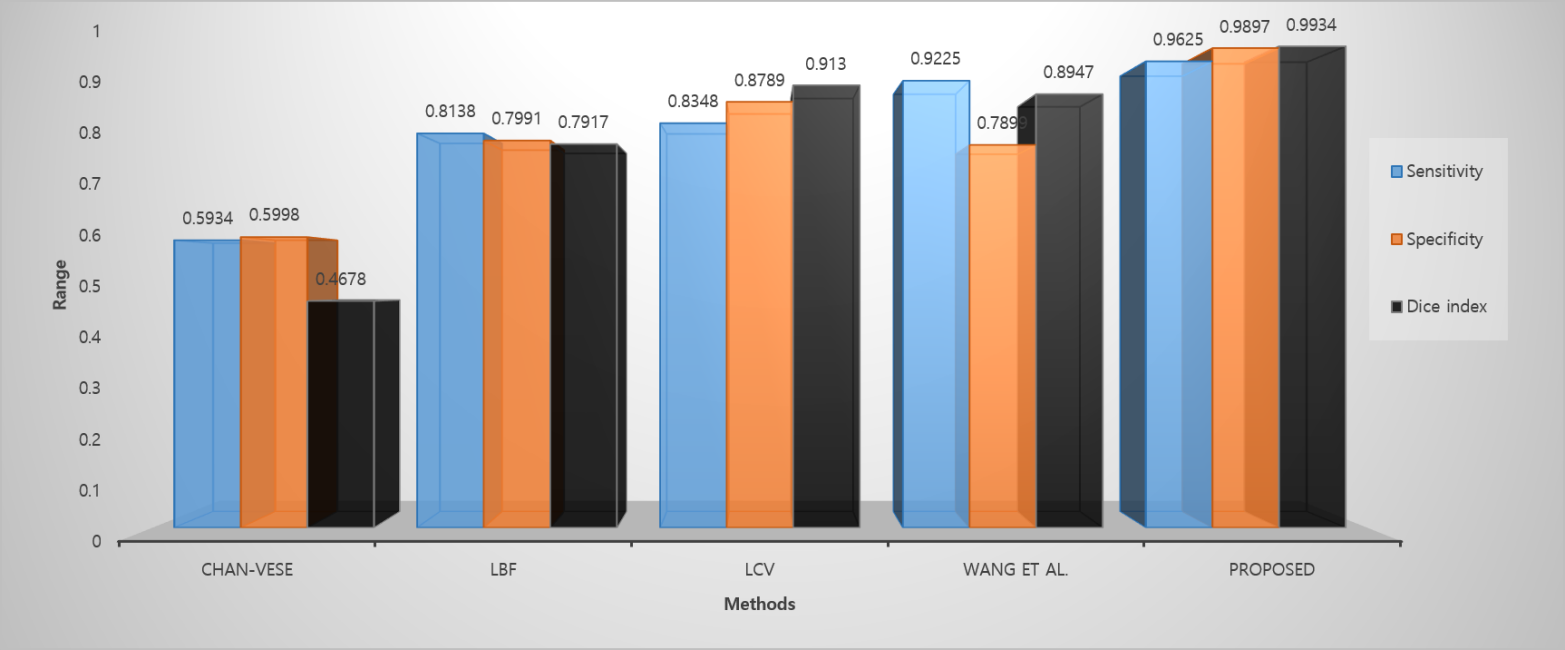
Comparative analysis of BRATS 2015 challenge dataset in terms of sensitivity, specificity and Dice index metrics.

Fig 11 summarize the comparative results of each method on BRATS 2015 challenge dataset, we compute the average sensitivity, specificity, and Dice index of each method against the manual ground truth. It shows that proposed method has demonstrated the capability and best rate of segmentation. The Chan-Vese method could not manage to get desired results, LBF and LCV methods have good Dice index values but their sensitivity and specificity values are not satisfactory, Wang et al method perform better with values close to the ground truth in some cases however it is unable to delineate proper boundaries in majority cases finally, proposed method on the other hand consistently outperformed traditional methods and achieved good results.

## Conclusion

In this paper, we have presented a hybrid novel two-stage segmentation method with application to synthetic and real (including medical) images combining global, local and edge intensity information. The first stage gets the intensity homogeneous segmentation result by employing global and edge information, after reaching to maximum number of iterations in the first stage we get the contour near the object boundaries. The second stage takes the final contour from the first stage and employs the local intensity and edge information to get the final accurate result for intensity inhomogeneous image segmentation. By using the concept of the two stages, proposed method provides an appropriate solution to the contour initialization which is a very common problem in traditional local active contour methods. Proposed method scales global and local region terms with edge function which prevents the boundary leakage and over segmentation problem. Finally, a Gaussian kernel is used to regularize the level set function and to avoid an expensive reinitialization.

The main contribution of our method is the formulation of the local and geodesic energy functional based on ESRAC [20] method for capturing intensity inhomogeneous regions. Initially, experimental results have been taken from a set of synthetic and real images in order to show the robustness of the proposed method. Finally, for qualitative and quantitative analysis we have evaluated the proposed method on publically available databse *PH*^2^ [28] for skin lesion and BRATS 2015 [27] for brain tumor segmentation. The comparative analysis shows that proposed method has obtained better results than previous state-of-art methods.

## Supporting information

S1 Dataset Contains synthetic and real images used in Figs 1, 3, 4, 5 and 6 (RAR). (ZIP)

## References

[1] El-Baz A, Acharya R, Mirmehdi M, Suri jasjit. Multi modality state-of-the-art medical image segmentation and registration methodologies. Springer Science & Business Media 2011(1)235–280.

[2] Tian Y, Duan F, Mingquan Z, Wu Z. Active contour model combining region and edge information. intMachine vision and applications. 2013 24(1)(47–61). doi: 10.1007/s00138-011-0363-7

[3] Kass M, Witkin A, Terzopoulos D. Snakes: Active contour models. international journal of computer vision. 1988 1(4)(321–331). doi: 10.1007/BF00133570

[4] Li C, Xu C, Gui C, Fox MD. Level set evolution without re-initialization: a new variational formulation. IEEE Computer Society Conference on Computer Vision and Pattern Recognition (CVPR’05). 2005 1:430–436.

[5] Caselles V, Kimmel R, Sapiro G. Geodesic active contours. International journal of computer vision. 1997 22(1):61–79. doi: 10.1023/A:1007979827043

[6] Caselles V, Catté F, Coll T, Dibos F. A geometric model for active contours in image processing. Numerische mathematik. 1993 66(1):1–33. doi: 10.1007/BF01385685

[7] Appleton B, Talbot H. Globally optimal geodesic active contours. Journal of Mathematical Imaging and Vision. 2005 23(1):67–86. doi: 10.1007/s10851-005-4968-1

[8] Yezzi A, Kichenassamy S, Kumar A, Olver P, Tannenbaum A. A geometric snake model for segmentation of medical imagery. IEEE Transactions on medical imaging. 1997 16(2):16–02. doi: 10.1109/42.563665 10.1109/42.563665 9101329

[9] Xiang Y, Chung AC, Ye J. An active contour model for image segmentation based on elastic interaction. Journal of computational physics. 2006 219(1):455–476. doi: 10.1016/j.jcp.2006.03.026

[10] Mumford D, Shah J. Optimal approximations by piecewise smooth functions and associated variational problems. Communications on pure and applied mathematics. 1989 42(5):577–685. doi: 10.1002/cpa.3160420503

[11] Lie J, Lysaker M, Tai XC. A binary level set model and some applications to Mumford-Shah image segmentation. IEEE Transactions on Image Processing. 2006 5(15):1171–1181. doi: 10.1109/TIP.2005.863956 10.1109/tip.2005.863956 16671298

[12] Chan TF, Vese LA. Active contours without edges. IEEE Transactions on image processing. 2001 10(2):266–277. doi: 10.1109/83.902291 18249617 10.1109/83.902291

[13] Li C, Kao CY, Gore JC, Ding Z. Implicit active contours driven by local binary fitting energy. IEEE Conference on Computer Vision and Pattern Recognition. 2007:1–7.

[14] Li C, Kao CY, Gore JC, Ding Z. Minimization of region-scalable fitting energy for image segmentation. IEEE transactions on image processing. 2008 17(10):1940–1949. doi: 10.1109/TIP.2008.2002304 18784040 10.1109/TIP.2008.2002304 PMC2720140

[15] Li C, Huang R, Ding Z, Gatenby JC, Metaxas DN, Gore JC. A level set method for image segmentation in the presence of intensity inhomogeneities with application to MRI. IEEE Transactions on Image Processing. 2011 20(07):2007–2016. doi: 10.1109/TIP.2011.2146190 21518662 10.1109/TIP.2011.2146190 PMC6952214

[16] Lankton S, Tannenbaum A. Localizing region-based active contours. IEEE transactions on image processing. 2008 17(11):2029–2039. doi: 10.1109/TIP.2008.2004611 18854247 10.1109/TIP.2008.2004611 PMC2796112

[17] Zhang K, Zhang L, Song H, Zhou W. Active contours with selective local or global segmentation: a new formulation and level set method. Image and Vision computing. 2010 28(04):668–679. doi: 10.1016/j.imavis.2009.10.009

[18] Wang L, Li C, Sun Q, Xia D, Kao CY. Active contours driven by local and global intensity fitting energy with application to brain MR image segmentation. Computerized Medical Imaging and Graphics. 2009 33(07):520–531. doi: 10.1016/j.compmedimag.2009.04.010 19482457 10.1016/j.compmedimag.2009.04.010

[19] Zhang L, Peng X, Li G, Li H. A novel active contour model for image segmentation using local and global region-based information. Machine Vision and Applications. 2016:1–16.

[20] Oh J, Martin DR, Hu X. Partitioned edge-function-scaled region-based active contour (p-ESRAC): Automated liver segmentation in multiphase contrast-enhanced MRI. Medical physics. 2014 41(04). doi: 10.1118/1.4867865 10.1118/1.4867865 24694145

[21] Wang H, Huang TZ, Xu Z, Wang Y. A two-stage image segmentation via global and local region active contours. Neurocomputing. 2016 (205):130–140.

[22] Soomro S, Akram F, Kim JH, Soomro TA, Choi KN. Active Contours Using Additive Local and Global Intensity Fitting Models for Intensity Inhomogeneous Image Segmentation. Computational and Mathematical Methods in Medicine. 2016 doi: 10.1155/2016/9675249 27800011 10.1155/2016/9675249 PMC5075425

[23] Soomro S, Akram F, Munir A, Lee CH, Choi KN. Segmentation of Left and Right Ventricles in Cardiac MRI Using Active Contours. Computational and Mathematical Methods in Medicine. 2017 doi: 10.1155/2017/8350680 28928796 10.1155/2017/8350680 PMC5591936

[24] Zhu S, Liu C, Wu Q Binary level set methods for topology and shape optimization of a two-density inhomogeneous drum. Computer Methods in Applied Mechanics and Engineering, 2010 199: 2970–2986. doi: 10.1016/j.cma.2010.06.007

[25] Liu C, Zhu S. A Semi-implicit binary level set method for source reconstruction problems. International Journal of Numerical Analysis & Modeling, 2011 8: 410–426.

[26] Liu C, Dong F, Zhu S, Kong D, Liu K. New variational formulations for level set evolution without reinitialization with applications to image segmentation. Journal of Mathematical Imaging and Vision, 2011 41: 194–209. doi: 10.1007/s10851-011-0269-z

[27] Menze BH, Jakab A, Bauer S, Kalpathy CJ, Farahani K, Kirby J, et al The multimodal brain tumor image segmentation benchmark (BRATS). IEEE transactions on medical imaging. 2015 (34)10:1993–2024. doi: 10.1109/TMI.2014.2377694 25494501 10.1109/TMI.2014.2377694 PMC4833122

[28] Mendonça T, Ferreira PM, Marques JS, Marcal A RS, Rozeira J. PH 2-A dermoscopic image database for research and benchmarking. Engineering in Medicine and Biology Society (EMBC), 2013 35th Annual International Conference of the IEEE. 2013:5437–5440. 10.1109/EMBC.2013.6610779 24110966

[29] Aubert G, Kornprobst P. Mathematical problems in image processing: partial differential equations and the calculus of variations. Springer Science & Business Media 2006(147).

[30] Balch CM, Buzaid AC, Soong SJ, Atkins MB, Cascinelli N, Coit DG, et al Final version of the American Joint Committee on Cancer staging system for cutaneous melanoma. Journal of Clinical Oncology. 2001 19(16):3635–3648. 11504745 10.1200/JCO.2001.19.16.3635

[31] Bauer S, Wiest R, Nolte LP, Reyes M. A survey of MRI-based medical image analysis for brain tumor studies. Physics in medicine and biology. 2013 58(13):R97 doi: 10.1088/0031-9155/58/13/R97 23743802 10.1088/0031-9155/58/13/R97

[32] Ilunga-Mbuyamba E, Avina-Cervantes JG, Garcia-Perez A, de Jesus Romero TR, Aguirre RH, Cruz AI, et al Localized active contour model with background intensity compensation applied on automatic MR brain tumor segmentation. Neurocomputing. 2017 220:84–97. doi: 10.1016/j.neucom.2016.07.057

