# Supplementary figures and images for: Hybrid two-stage active contour method with region and edge information for intensity inhomogeneous image segmentation
Source: PLoS One. 2018 Jan 29;13(1):e0191827. doi: 10.1371/journal.pone.0191827 (PMC5788363; doi:10.1371/journal.pone.0191827)

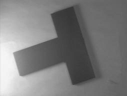

Supplement: S1 Dataset — (ZIP) [file pone.0191827.s001.zip › Oiginal images used/3.bmp]

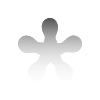

Supplement: S1 Dataset — (ZIP) [file pone.0191827.s001.zip › Oiginal images used/4.bmp]

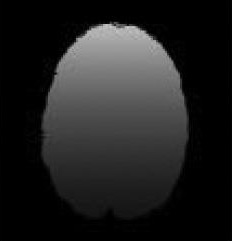

Supplement: S1 Dataset — (ZIP) [file pone.0191827.s001.zip › Oiginal images used/5.jpg]

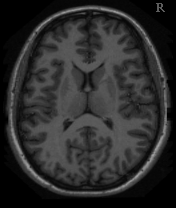

Supplement: S1 Dataset — (ZIP) [file pone.0191827.s001.zip › Oiginal images used/7.gif]

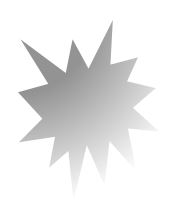

Supplement: S1 Dataset — (ZIP) [file pone.0191827.s001.zip › Oiginal images used/fig1.JPG]

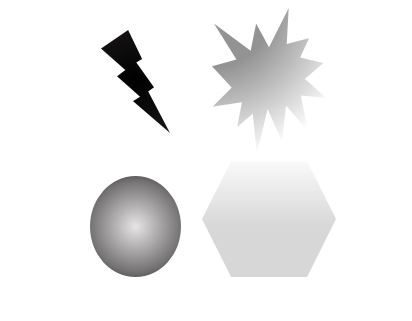

Supplement: S1 Dataset — (ZIP) [file pone.0191827.s001.zip › Oiginal images used/img3.JPG]

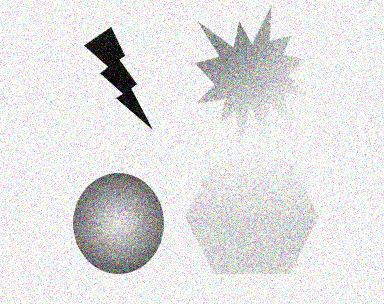

Supplement: S1 Dataset — (ZIP) [file pone.0191827.s001.zip › Oiginal images used/img3noisy.JPG]

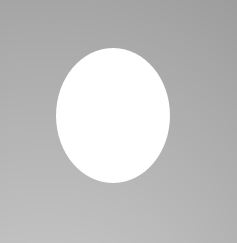

Supplement: S1 Dataset — (ZIP) [file pone.0191827.s001.zip › Oiginal images used/img4.JPG]

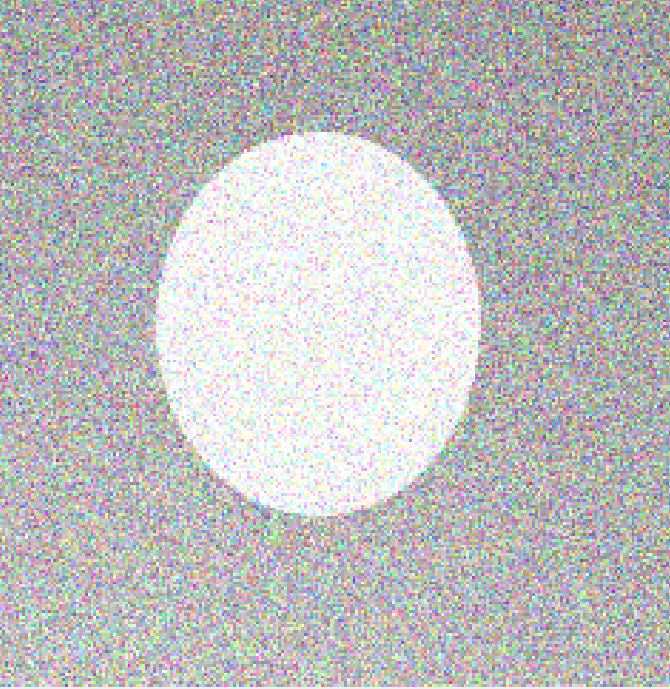

Supplement: S1 Dataset — (ZIP) [file pone.0191827.s001.zip › Oiginal images used/img4noisy.JPG]

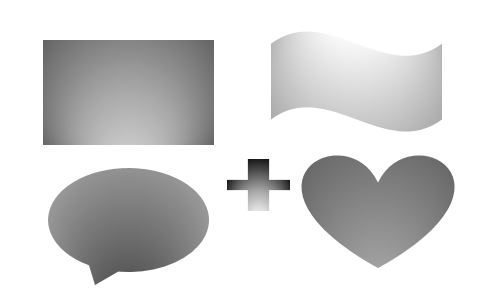

Supplement: S1 Dataset — (ZIP) [file pone.0191827.s001.zip › Oiginal images used/img5.JPG]

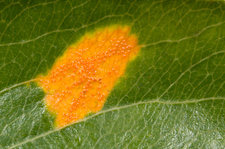

Supplement: S1 Dataset — (ZIP) [file pone.0191827.s001.zip › Oiginal images used/O19.jpg]

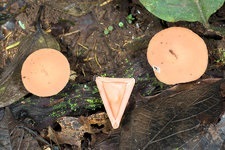

Supplement: S1 Dataset — (ZIP) [file pone.0191827.s001.zip › Oiginal images used/O20.jpg]

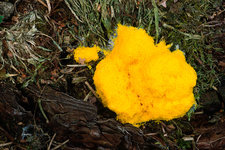

Supplement: S1 Dataset — (ZIP) [file pone.0191827.s001.zip › Oiginal images used/O21.jpg]

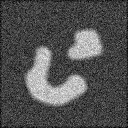

Supplement: S1 Dataset — (ZIP) [file pone.0191827.s001.zip › Oiginal images used/TwoObj.bmp]

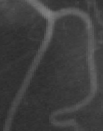

Supplement: S1 Dataset — (ZIP) [file pone.0191827.s001.zip › Oiginal images used/v.bmp]
